# Measuring decision quality: psychometric evaluation of a new instrument for breast cancer surgery

**DOI:** 10.1186/1472-6947-12-51

**Published:** 2012-06-08

**Authors:** Karen R Sepucha, Jeffrey K Belkora, Yuchiao Chang, Carol Cosenza, Carrie A Levin, Beverly Moy, Ann Partridge, Clara N Lee

**Affiliations:** 1 General Medicine Division, Massachusetts General Hospital, 50 Staniford Street, 9th floor, Boston, MA, 02114, USA; 2 Harvard Medical School, Boston, MA, USA; 3 Institute for Health Policy Studies, University of California, San Francisco, CA, USA; 4 Center for Survey Research, University of Massachusetts, 100 Morrissey Boulevard, Boston, MA, USA; 5 Informed Medical Decision Foundation, 40 Court Street, Boston, MA, USA; 6 Massachusetts General Hospital Cancer Center, 55 Fruit Street, Boston, MA, USA; 7 Dana-Farber Cancer Institute, Brigham and Women’s Hospital, Boston, MA, USA; 8 Division of Plastic and Reconstructive Surgery, Lineberger Comprehensive Cancer Center, Sheps Center for Health Services Research, University of North Carolina, CB Box 7195, Chapel Hill, NC, 27599-7195, USA

## Abstract

**Background:**

The purpose of this paper is to examine the acceptability, feasibility, reliability and validity of a new decision quality instrument that assesses the extent to which patients are informed and receive treatments that match their goals.

**Methods:**

Cross-sectional mail survey of recent breast cancer survivors, providers and healthy controls and a retest survey of survivors. The decision quality instrument includes knowledge questions and a set of goals, and results in two scores: a breast cancer surgery knowledge score and a concordance score, which reflects the percentage of patients who received treatments that match their goals. Hypotheses related to acceptability, feasibility, discriminant validity, content validity, predictive validity and retest reliability of the survey instrument were examined.

**Results:**

We had responses from 440 eligible patients, 88 providers and 35 healthy controls. The decision quality instrument was feasible to implement in this study, with low missing data. The knowledge score had good retest reliability (intraclass correlation coefficient = 0.70) and discriminated between providers and patients (mean difference 35%, p < 0.001). The majority of providers felt that the knowledge items covered content that was essential for the decision. Five of the 6 treatment goals met targets for content validity. The five goals had moderate to strong retest reliability (0.64 to 0.87). The concordance score was 89%, indicating that a majority had treatments concordant with that predicted by their goals. Patients who had concordant treatment had similar levels of confidence and regret as those who did not.

**Conclusions:**

The decision quality instrument met the criteria of feasibility, reliability, discriminant and content validity in this sample. Additional research to examine performance of the instrument in prospective studies and more diverse populations is needed.

## Background

The majority of patients diagnosed with early stage breast cancer are eligible to decide between having a mastectomy or lumpectomy with radiation (breast conserving therapy), yet this decision can be very challenging. Guidelines and consensus statements for breast cancer treatment emphasize the equivalence of mastectomy and breast conserving therapy for survival [1-3]. However, these options differ on other dimensions that matter to patients, particularly regarding appearance, need for radiation, likelihood of re-excision and risk of local recurrence.

After many years of increasing breast conserving surgery rates, several recent studies suggest that mastectomy rates are rising [4,5]. Proposed reasons for the rise have focused on different factors such as increased use of MRI imaging, younger patient age and patient preference. Rates of breast conserving surgery or rates of mastectomy have been proposed as quality measures for breast surgery; however, both of these measures have been found to be lacking [6,7]. In this situation, where survival is equivalent, the “best” treatment is dependent upon how each individual patient weighs the other factors, including cosmetic results, likelihood of recurrence and concerns about radiation. A measure that reports on high or low utilization does not provide any evidence that the right procedure is being used on the right patient.

For many situations, including surgery for early breast cancer, the appropriateness of surgery cannot be determined solely based on clinical factors [8,9]. An international consensus process including clinicians, patients and decision making experts endorsed a definition of decision quality that focuses on two key areas: (1) the extent to which patients are informed and (2) the extent to which treatments match what is most important to patients [10]. This definition of decision quality was taken as the basis for the development of a survey instrument to measure patients’ knowledge and the match between their goals and type of breast cancer surgery received.

In breast cancer, a few studies have used knowledge questionnaires to assess how informed breast cancer patients were about the surgery decision [11-14]. Other studies have examined patients’ goals and found that cosmetic concerns and concerns about recurrence play a big role in patients’ decisions, in addition to fear of radiation and desire to follow the doctors’ recommendation [15-19]. Each of these studies used different survey instruments and none of the studies examined both knowledge and extent to which patients’ goals were aligned with treatment. In summary, there is a lack of comprehensive, well validated instruments to measure the quality of surgical decisions in breast cancer.

To address this gap, a patient-reported survey instrument for early stage invasive breast cancer surgery decisions was developed to assess the extent to which patients were informed and received treatments that matched their goals. It is important that patient reported surveys demonstrate both strong psychometric properties (e.g., reliability and validity) and clinical sensibility (e.g., acceptability and feasibility) [20]. In this study, we examined performance of the Breast Cancer Surgery Decision Quality Instrument (BCS-DQI) along these criteria using three study samples: (1) breast cancer patients who had made a surgical treatment decision two or three years prior to the survey (2) breast cancer health care providers and (3) a group of healthy controls who had never had breast cancer. These samples provide complementary data on the performance of the instrument. The retrospective patient sample provided an “experienced” sample to evaluate the items and to examine stability of the responses. The provider sample was used to examine content validity and discriminant validity of the knowledge score. The healthy control sample was also used to examine discriminant validity of the knowledge score.

## Methods

### Approach and instrument development

The BCS-DQI has two decision-specific sections: (1) a set of knowledge questions to determine whether patients are informed and (2) a set of goals and concerns that are used in a model to determine the concordance, or extent to which patients receive treatments that reflected what is most important to them.

The definition of decision quality used to guide the instrument was validated by the International Patient Decision Aid Standards group [10]. The approach to instrument development was based on a conceptual framework of shared decision making by Mulley [21] and extended by Sepucha and Mulley [22] and Sepucha et al [23]. This framework takes a systems view of decision making and draws from both normative and behavioral decision theories. Both theories assume that people are goal-directed and will make choices that are in their best interest. Normative theories have fairly strict assumptions of pure rationality, whereas our approach draws from behavioural theories that recognize limitations (cognitive capacity, competing interests, biases and heuristics) that may result in deviations from rational choice. In addition, normative theories only consider utilities for health states as appropriate influences on choices, we have relaxed this to include other factors (e.g. the recovery time or number of surgeries) that may also appropriately influence choices. These factors are more generally referred to as “goals and concerns.”

The process for developing the BCS-DQI and preliminary validation work has been described in detail elsewhere [24,25]. The key content for the items was derived from the conceptual framework with significant input from breast cancer survivors and multidisciplinary group of clinical experts. The content of the knowledge items and goals was selected to cover core areas, but it was not meant to be exhaustive. For example, we did not include all factors that might influence patients’ preferred treatment, such as spouse’s goals, and instead focused on how patients felt about key tradeoffs for the two main options. Experts in survey research drafted multiple choice and open-ended items that covered the content. The draft surveys were subjected to cognitive testing with breast cancer survivors (n = 6), during which respondents completed the instrument while talking out loud to explain their thinking and interpretation of the instructions, items, and responses. Based on those results, minor revisions were made to improve acceptability and comprehension of the instruments.

### Sample and procedures

Adult women patients diagnosed in 2005 or 2006 with early stage (Stage 1 or 2) breast cancer were identified through cancer registries at four participating sites. Exclusion criteria (e.g. bilateral breast cancer, recurrent breast cancer, prior radiation) were set to ensure that the majority of patients were clinically eligible for both mastectomy and lumpectomy. Each patient’s treating physician had to approve contact. All patients received the study packet in the mail, and non-respondents received a reminder phone call after two weeks and a reminder packet after four weeks. Respondents received a small incentive with the initial packet and a small compensation (e.g., book of stamps) for completing the survey. A subset of patient respondents across all sites was sent a second, identical survey by mail approximately four weeks after responding, to assess test-retest reliability.

All health care professionals (surgeons, radiation oncologists, medical oncologists, plastic surgeons and oncology nurses) who work with breast cancer patients at the participating sites were invited to complete the knowledge portion of the decision quality instruments. Providers received the survey by mail. Non-responders received an email reminder after two weeks and a reminder mailing after four weeks. Provider respondents received $50 cash or a gift card upon completion of the survey.

Healthy controls who did not have a history of breast cancer or a first degree relative (mother, sister or daughter) with breast cancer and who were not health care providers, were recruited through email bulletins sent to all employees at one of the sites. Women were screened by phone and eligible women were mailed the surveys. Non-respondents received a reminder phone call after two weeks and a reminder mailing after four weeks. Respondents received $10 for completing the survey.

The study was approved by and conducted in accordance with the policies set out by the participating sites’ Institutional Review Boards.

### Measures

Data on treatment stage was taken from the cancer registry.

#### Patient survey

Patients reported standard demographics and breast cancer treatments.

Breast Cancer Surgery Decision Quality Instrument (BCS-DQI): included two main parts (1) 13 multiple choice and 2 open-ended knowledge items and (2) 8 goals and concerns rated on an 11-point scale from 0 (not at all important) to 10 (extremely important).

Other items were included in the patient survey to assess the following aspects of the decision:

Top three goals and concerns: patients selected their top three goals and concerns from those included in the BCS-DQI.

Patients preferred treatment: a single item asked, “Which treatment was your personal preference?” with possible responses of *Mastectomy*, *Lumpectomy and Radiation* or *I am not sure.*

Treatment received: was defined as the final surgical treatment received.

Decision confidence: assessed with one item, “On a scale of 0 to 10 where 0 is not at all confident and 10 is extremely confident, how confident are you that the decision about breast surgery was the right one for you?”

Decision regret: assessed with one item, “If you had the chance to make the decision again, would you have the same type of surgery? Definitely yes, probably yes, not sure, probably no, definitely no”.

#### Provider survey

Providers completed the knowledge portion of the BCS-DQI, and indicated the importance of each knowledge item on a scale of 1 (not important) to 4 (essential) and how well the overall set of items covered the essential information on a scale of 1 (not at all well) to 4 (extremely well).

#### Healthy control survey

Participants completed the knowledge portion of the BCS-DQI and some demographic items.

### Analysis

#### Sample evaluation

First, the sample characteristics were compiled to see how well they matched the sampling plan. Two-sample t-tests and chi-square tests were used to compare the patient characteristics between responders and non-responders. Chi-square tests were used to compare the response rate among sites. We also examined the interaction between patient characteristics and response rate by site. Additionally, two-sample t-tests and chi-square tests were used to compare demographics between patients and healthy volunteers. Next, the item non-response was examined.

#### Item retention and deletion

Items were examined for issues such as difficulty (e.g. knowledge items that were too easy, where healthy volunteers scored >80% or too hard, where provider average scores <50%), problematic format (if >5% had problems such as two responses check off when only one was expected), redundancy (items that had very high >0.8 inter-item correlation), and floor or ceiling effects (scores at top or bottom of the range for knowledge scores or goals). [26,27] The data were discussed by a steering group of six people that included experts in survey research, decision sciences and clinical experts in breast cancer (including three of the authors KS, CL, CC). Problematic items were deleted or revised.

#### BCS-DQI knowledge score

Each item received one point for correct response. A mean knowledge score was standardized by dividing the number of correct responses by the number of items, resulting in scores ranging from 0% to 100%. Open-ended items were scored correct if they fell within a range that was pre-determined by medical experts based on clinical evidence. Items with multiple parts were weighted to sum to one. The response, “I am not sure,” was considered incorrect. We scored missing responses as 1/k where k was the number of possible responses to the item (essentially equivalent to guessing). A knowledge score was calculated for every respondent who completed at least 50% of the items.

#### BCS-DQI concordance score

There is no standard approach to measuring concordance between patients’ goals and treatments [28]. The approach follows that used by Barry et al (1995) to examine the extent to which patients’ goals are associated with treatments [29]. We examined whether having mastectomy was associated with stage of disease and each of the goals both in univariate analyses, using t-tests for continuous variables and Chi-squared tests for categorical variables, and in multivariable analysis using a logistic regression model with treatment (mastectomy vs. lumpectomy) as the dependent variable. For the regression model, the missing responses from the goal items were imputed from the other available goal items using the EM algorithm [30].

We used the regression model to determine the model predicted probability of mastectomy for each patient. Patients with a predicted probability ≥0.5 who had mastectomy and those with a predicted probability <0.5 who had lumpectomy, were classified as concordant. A summary concordance score was calculated that indicated the percentage of patients whose decisions “matched” their goals. The score reflects how well the model fits the observed data. The interpretation of the score is at the group level. Groups with higher scores are better at matching treatments to patients’ goals. Patients who had lumpectomy followed by mastectomy were excluded from the concordance model as they were not clinically eligible for both options. Clinical considerations (e.g. inability to get clear margins) appropriately guided treatment for these patients, as a result, these patients might look like their goals are mismatched from their final treatment, mastectomy.

#### BCS-DQI screener

A short version of the DQI that includes 5 of the knowledge items and 4 of the goals was also created and scored in comparable way to the longer version. All of the goals that were significant in the multivariate concordance model are included in the screener version as well as concerns about reducing chance of having cancer come back in the breast.

#### Acceptability and feasibility

Acceptability was examined using length of time to complete the instrument, which was self-reported by patients, and response rates. Feasibility was examined using rates of missing data, with any item with more than 5% missing responses considered problematic and subject to revision. We examined association between average time to complete and missing responses by education level using ANOVA.

#### Assessment of reliability

Reproducibility, or test-retest reliability, for the full knowledge score and the screener knowledge scores and the individual goals was calculated using intraclass correlation coefficient (ICC). The *a priori* target was to exceed 0.7. [27] The knowledge items do not draw from a single underlying construct; as a result, we do not report Cronbach’s alpha or internal consistency.

#### Assessment of validity

There is no gold standard for knowledge score, goals and concerns, or concordance score. We tested several hypotheses, developed *a priori*, to provide evidence of validity:

(1) Content validity hypotheses:

a. For knowledge: More than 70% of providers will report that the knowledge items covered the key content very or extremely well.

b. For goals and concerns: At least 20% of patients will include the item as one of their top three issues for the decision.

(2) Discriminant validity hypotheses:

a. Providers would be more knowledgeable than patients, who in turn would be more knowledgeable than the healthy controls (tested using analysis of variance (ANOVA) with planned comparisons). This was tested for the full version and the screener version.

b. The individual goals and concerns will discriminate significantly between those who have mastectomy and those who have lumpectomy (tested using one-sided t-tests). For example, patients who rate the importance of “avoiding radiation” highly should be more likely to have mastectomy (because that option provides the highest chance of avoiding radiation).

c. Patients who stated a preference for mastectomy will have higher model predicted probabilities than those who were unsure, who in turn will have higher predicted probabilities than those who had a preference for lumpectomy and radiation (tested using ANOVA with planned comparisons).

(3) Predictive validity for concordance score.

a. Patients who received treatment that matched that predicted by the regression model will have higher confidence and less regret than those who did not (tested using two sided t-test and Chi-square test).

## Results

### Response rates and sample

The overall patient response rate was 60%. The eligible patient sample is described in Table 1. Responders only differed from non-responders on two factors, race/ethnicity and site. Responders were more likely to be white than non-responders (85% vs. 71%, p < 0.001). Response rates also varied significantly by site (ranged from 49% to 70%, p < 0.001). We examined respondent characteristics by site, and only one site had signficant interactions. At that site, non-white race (37% vs. 61%, p = 0.001) and having hormonal therapy (39% vs. 57% for those who did and did not have hormonal therapy respectively, p = 0.02) were associated with a lower response rate.

**Table 1:** Demographic and treatment characteristics of patient and healthy control samples

	**Patients**		**Healthy controls**	
**Characteristic**	**N = 440**		**N = 35**	
Age in years, mean (SD)	56.9	(11.3)	42.4	(10.9)
Race, N (%)				
White	365	(83.0)	26	(74.3)
Black	35	(8.0)	3	(8.6)
Other	40	(9.1)	5	(14.3)
Education, N (%)				
High school or less	55	(12.5)	0	0
Some college	106	(24.1)	8	(22.9)
College graduate or more	279	(63.4)	27	(77.1)
Annual income, N (%)				
<30,000	59	(13.4)	1	(2.9)
30,000-59,999	87	(19.8)	11	(31.4)
60,000-100,000	107	(24.3)	14	(40.0)
>100,000	160	(36.4)	8	(22.9)
Marital status, N (%)				
Married/Partnered	297	(67.5)	23	(65.7)
Other	141	(32.0)	12	(34.3)
Months since diagnosis, mean (SD)	30.8	(9.8)	--	
Stage I (vs. II), N (%)	265	(60.2)	--	
Treatment, N (%)				
Mastectomy* (vs. Lumpectomy)	168	(38.2)	--	
Breast reconstruction	80	(18.2)	--	
Radiation	256	(58.2)	--	

*57 patients had lumpectomy initially, followed by mastectomy.

SD=standard deviation.

A majority of providers, 88/116 (79%), completed the survey. The provider sample was on average 45 years old (SD 9), mostly female (65%), and in practice for 15 years (SD 11). The provider sample was 33% medical oncologists, 30% nurses (including oncology nurses, nurse practitioners, and registered nurses), 17% general surgeons and surgical oncologists, 8% radiation oncologists and 8% plastic surgeons. The response rate for the healthy controls was 100%. Healthy controls were significantly younger than the patient sample (mean age 42 versus 57, p < 0.001) but did not vary significantly from the patient sample on any other demographic variables.

### Item retention and deletion

**Figure 1:**
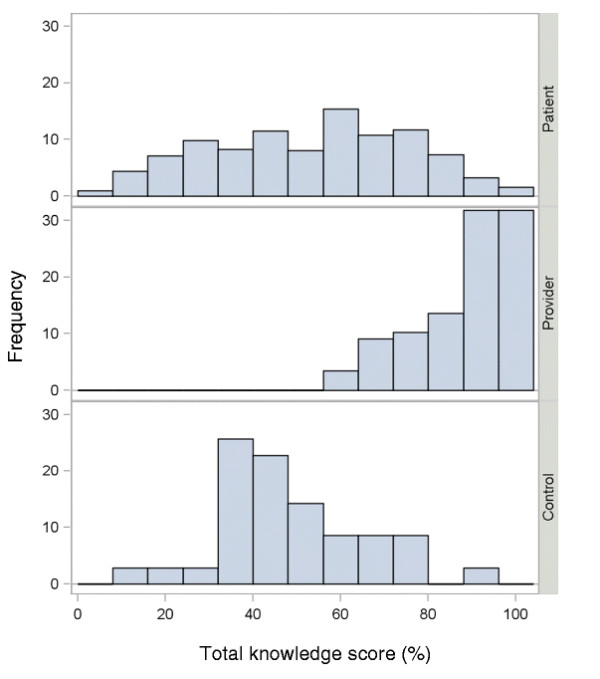
Knowledge score distributions for patients, providers and healthy controls.

The total knowledge scores ranged from 0-100% and there was no evidence of a floor or ceiling effect, i.e. scores were not concentrated at the bottom or top of the scale (see Figure 1). Two knowledge items were deleted for being too easy and one was deleted for negative item to total correlation. The reduced set of items was used in the remaining analyses. For each goal and concern, the responses spanned the entire range of possibilities (0 to 10). One item, “avoid cancer coming back in the breast,” had evidence of a ceiling effect as 88% of respondents marked 10. All of the goals were used in the analyses.

### Acceptability and feasibility

The BCS-DQI took an average of 6 minutes for patients to complete (range 1.4-19.0 minutes). The time to complete was associated with education level, as patients with high school degree or less took longer than those with some college and those with college degree or more (6.8 vs. 6.4 vs. 5.3 minutes respectively, p < 0.001). The knowledge items had an average of 1.4% missing responses (range 0.2-2.5%). Four respondents (0.9%) did not complete enough of the knowledge items to calculate a total knowledge score. The goals and concerns had a median of 3.3% missing responses (range 0.9-10.9%). One goal was an outlier for missing responses, “remove breast for peace of mind,” with 10.9% missing. The majority of those who skipped it (94%) had had a lumpectomy. The number of missing responses was not associated with education level (p = 0.62).

### Reliability

The retest reliability of the knowledge score was intraclass correlation coefficient (ICC) = 0.70. The short (5-item) version of the knowledge score had lower reliability (ICC = 0.60). The retest reliability of the goals were: keep breast ICC = 0.87, remove breast ICC = 0.83, avoid radiation ICC = 0.76, avoid cancer coming back in the breast ICC = 0.66, avoid serious side effects of radiation ICC = 0.64, and avoid hassle of radiation ICC = 0.61.

### Validity of knowledge scores

The majority of providers (76.1%) felt that the set of items covered the key facts very or extremely well supporting content validity.

Patients’ mean total knowledge score was 52.7% (SD 21.8). Providers were significantly more informed than patients (87.7% vs. 52.7%, p < 0.001). Patients were more informed than healthy controls, but the difference was not significant (52.7% vs. 49.3%, p = 0.28). Figure 1 shows the overall distributions and Table 2 shows responses for selected knowledge items.

**Table 2:** Responses to selected knowledge items from the Breast Cancer Surgery Decision Quality Instrument

	**Patients N (%)**	**Healthy Volunteers N (%)**	**Providers N (%)**
For most women with early breast cancer, how much would waiting 4 weeks to make a treatment decision affect their chances of survival?			
A lot	57 (13)	4 (11)	1 (1)
Somewhat	62 (14)	16 (46)	1 (1)
A little or not at all*	263 (60)	13 (37)	85 (97)
Not sure	55 (13)	2 (6)	1 (1)
With treatment, about how many women diagnosed with early breast cancer will eventually die of breast cancer?			
Most will die of breast cancer	4 (1)	0	0
About half will die of breast cancer	16 (4)	9 (26)	1 (1)
Most will die of something else*	312 (71)	24 (69)	87 (99)
Not sure	106 (24)	2 (6)	0
After which treatment is it more likely that women will need to have another operation to remove the tumor?			
Lumpectomy*	304 (70)	30 (86)	88 (100)
Mastectomy	3 (1)	0	0
Equally likely for both	47 (11)	4 (11)	0
Not sure	78 (18)	1 (3)	0
On average, which women with early breast cancer live longer?			
Women who have a mastectomy	42 (10)	9 (26)	3 (3)
Women who have a lumpectomy and radiation	17 (4)	2 (6)	1 (1)
There is no difference*	245 (57)	11 (31)	84 (96)
Not sure	129 (30)	13 (37)	0
On average, which women have a higher chance of having cancer come back in the breast that has been treated?			
Women who have a mastectomy	1 (0.2)	0	0
Women who have a lumpectomy and radiation*	202 (46)	23 (68)	67 (77)
There is no difference	124 (28.5)	4 (12)	19 (22)
Not sure	108 (25)	7 (21)	1 (1)

*Indicates the correct answer.

The screener knowledge score (5-item) version was highly correlated with the total knowledge score (Pearson =0.80). It also had similar results with providers being significantly more informed than patients (93.6% vs. 60.7%, p < 0.001) and with patients having similar knowledge to the healthy controls (60.7% vs. 57.7%, p < 0.001).

### Validity of concordance score

Only one goal, *avoiding the hassle of radiation* did not meet the *a priori* cutoff of 20% for content validity (19% of lumpectomy patients and 12% of mastectomy patients selected it in their top three). All other goals had 30% or more patients select it as one of their top three issues.

In univariate analyses, five of the six goals significantly discriminated between the surgical options in the expected direction. In the multivariable logistic regression model, three goals were significantly associated with receipt of mastectomy: desire to keep breast, remove breast for peace of mind, and avoid radiation (see Table 3). The logistic regression model had a c statistic of 0.95, indicating very good predictive accuracy of the model. The concordance score, or percentage of patients who got the treatment predicted by the model, was 89%. Concordance did vary by treatment, as 82% of mastectomy patients were predicted correctly, compared to 93% of lumpectomy patients (p = 0.007).

**Table 3:** Univariate (t-test or chi-square) and multivariable logistic regression analyses predictors of having mastectomy

	**Mast**	**Lump**	**Univariate**	**Multivariable**
**Factor**	**N = 111**	**N = 272**	**p**	**OR (95% CI)**
Stage II (vs. I)	53.2	32.7	0.0002	1.81(0.89, 3.68)
Importance: (on a scale from 0 to 10)				
**Keep breast**	**3.0**	**6.6**	**<0.0001**	**0.79 (0.70, 0.88)**
**Remove breast for peace of mind**	**9.3**	**3.5**	**<0.0001**	**1.88 (1.60, 2.22)**
Avoid having cancer come back in the breast	9.9	9.6	0.0003	
**Avoid radiation**	**5.1**	**2.1**	**<0.0001**	**1.23 (1.11, 1.36)**
Avoid side effects of radiation	6.0	5.5	0.21	
Avoid hassle of radiation	4.4	2.4	<0.0001	

Factors in bold were significant in the multivariable regression model and used to calculate the concordance score.

Mast=mastectomy; Lump=lumpectomy; OR=odds ratio; CI=confidence interval.

The model discriminated well among patients who stated a preference for mastectomy, those who were unsure, and those who preferred lumpectomy (model predicted probabilities of 0.70 vs. 0.30 vs. 0.08, respectively, p < 0.0001 for all comparisons). Patients who “matched” had similarly high levels of confidence in their decision (9.12 vs. 9.07, p = 0.86) and were just as likely to want to do the same thing again (91% vs. 85%, p = 0.13) compared to those who did not match.

## Discussion

The purpose of the Breast Cancer Surgery Decision Quality Instrument is to provide a comprehensive assessment of the extent to which patients make informed decision and receive treatments that match their goals. The knowledge score had good retest reliability and was able to discriminate between providers and patients. The concordance score provides an assessment of how well treatments matched patients’ goals. The goals used in calculating the concordance score had good retest reliability. Patients were able to complete the BCS-DQI on their own, with low rates of missing items indicating that it was feasible.

The knowledge items cover content that the majority of providers feel is very important for patients to know before making a decision. Patients had some gaps in knowledge, e.g. only 57% correctly answering that survival is the same for mastectomy and lumpectomy with radiation. It was surprising that the knowledge scores of the healthy controls were similar to those of the breast cancer survivors. This result could be due to the fact that the controls were more knowledgeable generally (due to working in health care environment), or that survivors’ breast cancer knowledge had decreased over time. Other studies have found similarly low levels of knowledge for patients surveyed much closer to the time of diagnosis, which suggests that timing may not fully explain the lack of knowledge for this sample [14,24].

Three of the goals and concerns, importance of keeping the breast, removing the breast for peace of mind, and avoiding radiation, met the criteria for retest reliability and also discriminated between the options. The first two goals may appear to be simply two ends of the same issue, and although they are negatively correlated, they are not redundant (Pearson = −0.46). Similar concerns have been found to be related to treatment choices in other breast cancer studies [16,17,19,31]. Clinicians seeking to elicit patients’ preferences should, at a minimum, discuss how patients feel about keeping or losing their breast and how they feel about radiation.

The concordance score was high, indicating that most patients received treatments that matched their goals. Patients who preferred mastectomy were somewhat less likely to receive it (82%) compared to those who preferred lumpectomy (92%). Contrary to our hypotheses, we did not find evidence that respondents who received treatments that matched their goals had higher confidence or less decisional regret. This was possibly due to a ceiling effect with these items, as all patients had very high confidence and low regret. Several studies have found a positive relationship between decision making processes, such as being offered a choice of breast surgery and matching preferred level of involvement in decision making, with health outcomes including body image, psychological adjustment, and satisfaction [18,19,32,33]. It will be important to examine associated between concordance and these other outcomes in prospective studies.

The definition for concordance used here requires that the treatments received match patients’ goals. There are a growing number of studies that are reporting this metric, although the studies define and measure concordance differently [28]. The multidimensional measure of informed choice is one approach that combines knowledge and value concordance into a single measure [34]. It was developed to measure the quality of decision about prenatal testing and has been adapted for use in genetic testing for cancer [34-36]. The ability to reliably document that the treatments received, or the care delivered, reflects patients’ goals, needs and wants will be important.

The BCS-DQI was designed to audit the quality of decisions and to compare performance of providers or breast cancer centers on how well they inform their patients and how well they tailor treatments to patients’ goals. Clinicians have requested a short version that could be used in routine practice as a screening tool to assess patients’ knowledge and goals before the visit so that they could address any gaps. A short version, the BCS-DQI Screener, includes 5 knowledge items and 5 goals. Since there are fewer items, it might miss knowledge gaps or key goals that patients have. The purpose is to stimulate conversation between patients and providers about options, outcomes and goals, but not to limit content to only those items included.

Collins et al. (2009) surveyed newly diagnosed patients with an earlier version of the BCS-DQI Screener after patients had viewed a decision aid and before they saw their surgeon. They found high patient knowledge scores, for example, 98% of respondents answered the question about survival equivalence of the treatments correctly compared to 57% from the retrospective sample reported here [37]. In general, the patients’ knowledge scores in their study (86%) were comparable to the providers’ scores (87%). Further, in the Collins study, the same three goals were significant in the multivariable treatment model, suggesting the concordance model may hold for newly diagnosed patients who are actively making the treatment decision [37].

The current study has several limitations that should be noted. First, it was a retrospective study and patients were surveyed about 2 ½ years after the decision during which time their knowledge, goals and concerns likely changed. The survey relies on patient report and may not fully reflect what information was conveyed by clinicians during the decision making process. The sample was from four academic, National Cancer Institute-designated comprehensive cancer centers, and the performance may be different in community settings. Non-white patients had a lower response rate than white patients which raise questions about acceptability across diverse populations. Several studies have documented lower response rates and lower participation in research studies for non-white participants [38,39]. Further, the content validity was established on the full set of items and it is possible that the reduced set and the 5-item screener would not be reviewed as highly. It will be important to further test the BCS-DQI in order to understand the acceptability and performance of the instrument across more diverse populations and practice settings, and with patients at the time of the decision.

## Conclusions

The pressure on hospitals and health care providers to justify surgical treatments will continue to mount. Having a well validated, feasible survey instrument that can provide evidence that patients were informed and that the treatment reflected patients’ goals will be an important part of efforts to document quality of breast care. The BCS-DQI met several key criteria for high quality, patient-reported survey instruments including feasibility, retest reliability, discriminant validity and content validity, and may be useful in assessing the quality of breast cancer surgical decisions.

## Competing interests

Dr. Sepucha receives salary and research support from the not-for-profit Informed Medical Decision Foundation. Dr. Levin receives salary support as Research Director for the Informed Medical Decision Foundation, a not-for-profit (501(c)3) private foundation (http://www.informedmedicaldecisions.org). The Foundation develops content for patient education programs. The Foundation has an arrangement with a for-profit company, Health Dialog, to co-produce these programs. The programs are used as part of the decision support and disease management services Health Dialog provides to consumers through health care organizations and employers.

## Authors’ contributions

All authors contributed substantially to one or more of the studies including (1) the conception and design of patient study (KRS, JB, CC, BM, AP, CL) and provider study (KRS, JB, BM, AP), acquisition of data (KRS, JB, BM, AP), or analysis and interpretation of data (KRS, JB, YC, BM, AP, CL) (2) drafting the article or revising it critically for important intellectual content (all authors) (3) final approval of the version to be submitted (all authors). The corresponding author, KRS (ksepucha@partners.org), is responsible for the integrity of the work as a whole.

## Pre-publication history

The pre-publication history for this paper can be accessed here:

http://www.biomedcentral.com/1472-6947/12/51/prepub

## References

[B1] Fisher B Anderson S Bryant J Margolese RG Deutsch M Fisher ER Jeong JH Wolmark N Twenty-year follow-up of a randomized trial comparing total mastectomy, lumpectomy, and lumpectomy plus irradiation for the treatment of invasive breast cancer N Engl J Med 2002 347 1233 41 10.1056/NEJMoa022152 12393820

[B2] Early Breast Cancer Trialists’ Collaborative Group Effects of radiotherapy and surgery in early breast cancer–an overview of the randomized trials N Engl J Med 1995 333 1444 55 7477144 10.1056/NEJM199511303332202

[B3] Treatment of early-stage breast cancer Consensus statement, NIH Consensus Development Conference, June 18–21, 1990 (ed 8) 1990 National Institutes of Health, Bethesda, MD 2247093

[B4] Katipamula R Degnim AC Hoskin T Boughey JC Loprinzi C Grant CS Brandt KR Pruthi S Chute CG Olson JE Couch FJ Ingle JN Goetz MP Trends in mastectomy rates at the Mayo Clinic Rochester: effect of surgical year and preoperative magnetic resonance imaging J Clin Oncol 2009 25 4082 4088 19636020 10.1200/JCO.2008.19.4225 PMC2734420

[B5] McGuire KP Santillan AA Kaur P Meade T Parbhoo J Mathias M Shamehdi C Davis M Ramos D Cox CE Are mastectomies on the rise? A 13-year trend analysis of the selection of mastectomy versus breast conservation therapy in 5865 patients Ann Surg Oncol 2009 16 2682 90 10.1245/s10434-009-0635-x 19653046

[B6] McCahill LE Privette E James T Sheehey-Jones J Ratliff J Majercik D Krag DN Stanley M Harlow S Quality measures for breast cancer surgery: initial validation of feasibility and assessment of variation among surgeons Arch Surg 2009 144 455 462 10.1001/archsurg.2009.56 19451489

[B7] Lazovich D Solomon CC Thomas DB Moe RE White E Breast conservation therapy in the United States following the 1990 National Institutes of Health consensus development conference on the treatment of patients with early stage invasive breast carcinoma Cancer 1999 86 628 37 10.1002/(SICI)1097-0142(19990815)86:4<628::AID-CNCR11>3.0.CO;2-L 10440690

[B8] Lee CN Ko CY Beyond outcomes—the appropriateness of surgical care JAMA 2009 302 1580 81 10.1001/jama.2009.1465 19826028

[B9] Woloshin S Schwartz LM Invited commentary: early-stage breast cancer treatment for elderly women-does one size fit all? Surgery 2000 128 865 867 10.1067/msy.2000.109531 11056453

[B10] Elwyn G O'Connor A Stacey D Volk R Edwards A Coulter A Thomson R Barratt A Barry M Bernstein S Butow P Clarke A Entwistle V Feldman-Stewart D Holmes-Rovner M Llewellyn-Thomas H Moumjid N Mulley A Ruland C Sepucha K Sykes A Whelan T Developing a quality criteria framework for patient decision aids: online international delphi consensus process BMJ 2006 333 417 10.1136/bmj.38926.629329.AE 16908462 PMC1553508

[B11] Goel V Sawka CA Thiel EC Gort EH O’Connor AM Randomized trial of a patient decision aid for choice of surgical treatment for breast cancer Med Decis Making 2001 21 1 6 10.1177/0272989X0102100101 11206942

[B12] Street RL Voigt B Geyer CJ Manning T Swanson GP Increasing patient involvement in choosing treatment for early breast cancer Cancer 1995 76 2275 2285 10.1002/1097-0142(19951201)76:11<2275::AID-CNCR2820761115>3.0.CO;2-S 8635032

[B13] Whelan T Levine M Willan A Gafni A Sanders K Mirsky D Chambers S O’Brien MA Reid S Dubois S Effect of a decision aid on knowledge and treatment decision making for breast cancer surgery: a randomized trial JAMA 2004 292 435 441 10.1001/jama.292.4.435 15280341

[B14] Fagerlin A Lakhani I Lantz PM Janz NK Morrow M Schwartz K Deapen D Salem B Liu L Katz SJ An informed decision? Breast cancer patients and their knowledge about treatment Patient Educ Couns 2006 64 303 312 10.1016/j.pec.2006.03.010 16860523

[B15] Katz SJ Lantz PM Paredes-Alexander Y Janz NK Fagerlin A Liu L Deapen D Breast cancer treatment experiences of Latinas in Los Angeles County Am J Public Health 2005 95 2225 2230 10.2105/AJPH.2004.057950 16257945 PMC1449511

[B16] Molenaar S Oort F Sprangers M Rutgers E Luiten E Mulder J de Haes H Predictors of patients' choices for breast-conserving therapy or mastectomy: a prospective study Br J Cancer 2004 90 2123 30 15150557 10.1038/sj.bjc.6601835 PMC2409497

[B17] Temple WJ Russell ML Parsons LL Huber SM Jones CA Bankes J Eliaszew M Conservation surgery for breast cancer as the preferred choice: a prospective analysis J Clin Oncol 2006 24 3367 3373 10.1200/JCO.2005.02.7771 16849750

[B18] Mandelblatt JS Hadley J Kerner JF Schulman KA Patterns of breast carcinoma treatment in older women: patient preference and clinical and physician influences Cancer 2000 89 561 573 10.1002/1097-0142(20000801)89:3<561::AID-CNCR11>3.0.CO;2-A 10931455

[B19] Figueiredo MI Cullen J Hwang YT Rowland JH Mandelblatt JS Breast cancer treatment in older women: does getting what you want improve your long-term body image and mental health? J Clin Oncol 2004 22 4002 4009 10.1200/JCO.2004.07.030 15459224

[B20] Fitzpatrick R Davey C Buxton MJ Jones DR Evaluating patient-based outcome measures for use in clinical trials Health Technol Assess 1998 2 1 74 9812244

[B21] Mulley AJ Methodological issues in the application of effectiveness and outcomes research to clinical practice Effectiveness and Outcomes in Health Care 1999 National Academy Press, Washington D.C

[B22] Sepucha K Mulley A Extending decision support: preparation and implementation Patient Educ Couns 2003 50 269 271 10.1016/S0738-3991(03)00048-X 12900098

[B23] Sepucha K Ozanne E Mulley A Doing the right thing: systems support for decision quality in cancer care Ann Behav Med 2006 32 172 178 10.1207/s15324796abm3203_2 17107289

[B24] Sepucha K Ozanne E Silvia K An approach to measuring the quality of breast cancer decisions Patient Educ Couns 2007 65 261 269 10.1016/j.pec.2006.08.007 17023138

[B25] Lee C Barry M Cosenza C Development of instruments to measure the quality of breast cancer treatment decisions Health Expect 2010 13 258 272 20550591 10.1111/j.1369-7625.2010.00600.x PMC2919601

[B26] Fowler F Survey Research Methods, vol. 1 1993 Sage Publications, Inc, Thousand Oaks, CA

[B27] Nunnally J Bernstein I Psychometric Theory 1994 3 McGraw-Hill, New York

[B28] Sepucha K Ozanne EM How to define and measure concordancebetween patients’ preferences and medical treatments: a systematic review of approaches and recommendations for standardization Patient Educ Couns 2010 78 12 23 10.1016/j.pec.2009.05.011 19570647

[B29] Barry M Fowler FJ Mulley AJ Henderson JV Wennberg JE Patient reactions to a program designed to facilitate patient participation in treatment decisions for benign prostatic hyperplasia Med Care 1995 33 771 782 10.1097/00005650-199508000-00003 7543639

[B30] Dempster AP Laird NM Rubin DB Maximum likelihood from incomplete data via the EM algorithm J R Stat Soc, Ser B 1977 39 1 38

[B31] Stanton AL Estes MA Estes NC Cameron CL Danoff-Burg S Irving Lori M Treatment decision making and adjustment to breast cancer: a longitudinal study J Consult Clin Psychol 1998 66 313 322 9583334 10.1037//0022-006x.66.2.313

[B32] Andersen M Urban N Involvement in decision-making and breast cancer survivor quality of life Ann Behav Med 1999 21 201 209 10.1007/BF02884834 10626025

[B33] Keating N Guadagnoli E Landrum M Borbas C Weeks J Treatment decision making in early-stage breast cancer: should surgeons match patients' desired level of involvement? J Clin Oncol 2002 20 1473 1479 10.1200/JCO.20.6.1473 11896094

[B34] Michie S Dormandy E Marteau T The multidimensional measure of informed choice: a validation study Patient Edu Couns 2002 48 87 91 10.1016/S0738-3991(02)00089-7 12220754

[B35] Wakefield C Meiser B Homewood J Peate M Taylor A Lobb E Young MA Williams R Dudding T Tucker K The AGenDA Collaborative Group A randomized controlled trial of a decision aid for women considering genetic testing for breast and ovarian cancer risk Breast Cancer Res Treat 2008 107 289 301 10.1007/s10549-007-9539-2 17333332

[B36] Wakefield C Meiser B Homewood J Ward R O’DOnnel S Kirk J Australian GENtic testing Decision Aid Collaborative Group Randomized trial of a decision aid for individuals considering genetic testing for hereditary nonpolyposis colorectal cancer risk Cancer 2008 113 956 965 10.1002/cncr.23681 18618513

[B37] Collins ED Moore C Clay K O'Connor AM Llewellyn-Thomas HA Barth RJ Sepucha KR Can women make an informed decision for mastectomy? J Clin Oncol 2009 27 519 525 19114703 10.1200/JCO.2008.16.6215

[B38] Murthy VH Krumholz HM Participation in cancer clinical trials: Race-, sex-, and age-based disparities JAMA 2004 291 2720 2726 10.1001/jama.291.22.2720 15187053

[B39] Gross CP Filardo G Mayne ST Krumholz HM The impact of socioeconomic status and race on trial participation for older women with breast cancer Cancer 2005 103 483 491 10.1002/cncr.20792 15597407

